# Emerging Radioligands as Tools to Track Multi-Organ Senescence

**DOI:** 10.3390/diagnostics15192518

**Published:** 2025-10-04

**Authors:** Anna Gagliardi, Silvia Migliari, Alessandra Guercio, Giorgio Baldari, Tiziano Graziani, Veronica Cervati, Livia Ruffini, Maura Scarlattei

**Affiliations:** Nuclear Medicine Division, University Hospital of Parma, Via Antonio Gramsci 14, 43126 Parma, Italy; agagliardi@ao.pr.it (A.G.); smigliari@ao.pr.it (S.M.); alessandra.guercio@ao.pr.it (A.G.); gbaldari@ao.pr.it (G.B.); tgraziani@ao.pr.it (T.G.); vcervati@ao.pr.it (V.C.); mscarlattei@ao.pr.it (M.S.)

**Keywords:** senescence, biomarkers, age-related diseases, radiopharmaceuticals, PET imaging

## Abstract

Senescence is a dynamic, multifaceted process implicated in tissue aging, organ dysfunction, and intricately associated with numerous chronic diseases. As senescent cells accumulate, they drive inflammation, fibrosis, and metabolic disruption through the senescence-associated secretory phenotype (SASP). Despite its clinical relevance, senescence remains challenging to detect non-invasively due to its heterogeneous nature and the lack of universal biomarkers. Recent advances in the development of specific imaging probes for positron emission tomography (PET) enable in vivo visualization of senescence-associated pathways across key organs, such as the lung, heart, kidney, and metabolic processes. For instance, [^18^F]FPyGal, a β-galactosidase-targeted tracer, has demonstrated selective accumulation in senescent cells in both preclinical and early clinical studies, while FAP-targeted radioligands are emerging as tools for imaging fibrotic remodeling in the lung, liver, kidney, and myocardium. This review examines a new generation of PET radioligands targeting hallmark features of senescence, with the potential to track and measure the process, the ability to be translated into clinical interventions for early diagnosis, and longitudinal monitoring of senescence-driven pathologies. By integrating organ-specific imaging biomarkers with molecular insights, PET probes are poised to transform our ability to manage and treat age-related diseases through personalized approaches.

## 1. Introduction

Cellular senescence is a complex and multifaceted process characterized by a permanent halt in the cell cycle, which can diminish the regenerative capacity and functionality of tissues [1]. Senescence occurs throughout the lifespan, and the number of senescent cells increases with age, contributing to the normal aging process [2,3], while generating a higher risk for age-related disorders [4], including cancer [5], neurodegeneration [6,7], metabolic and cardiovascular diseases [8,9,10], chronic kidney disease [11], and inflammatory diseases [12]. Recent evidence suggests that the senescence response also drives degenerative processes such as organ-specific fibrosis [13] and the biological phenomenon underlying frailty [14] through the secretion of pro-inflammatory mediators [15].

**Figure 1:**
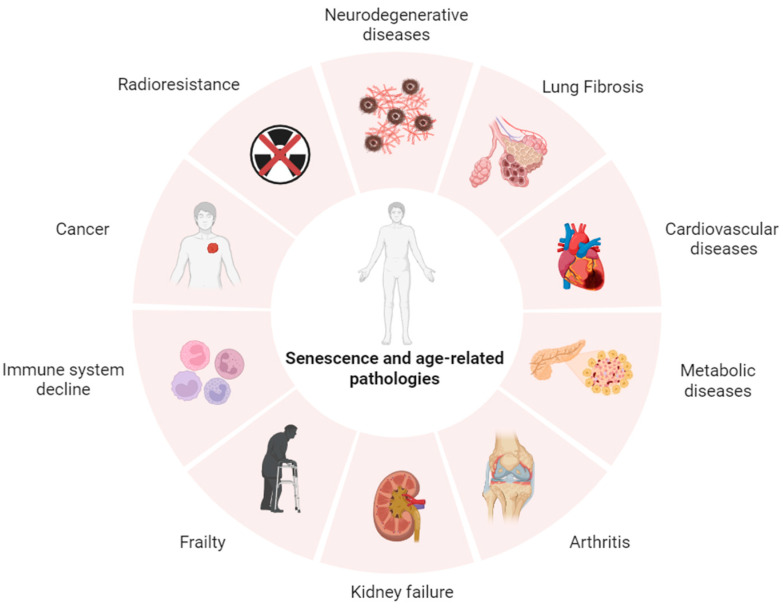
Cellular senescence contributes to aging and is involved in age-related diseases such as, among others, atherosclerosis, fibrosis, neurodegenerative disorders, and diabetes.

In cancer therapy, early senescence and production of senescence-associated cytokines represent major determinants of radioresistance [16,17], while in cancer survivors, cellular senescence contributes to accelerated aging and frailty [18]. Accumulation of senescent cells in the aging brain accelerate the aging processes and promote progression of tauopathies, including Alzheimer’s disease [19] (Figure 1).

Since life expectancy is increasing worldwide, understanding the mechanisms of senescence and aging has become increasingly critical for addressing age-related diseases. At the same time, complexity and heterogeneity of senescence phenotypes push the identification of non-invasive biomarkers for early detection and monitoring of the induced changes in organs and tissues.

Key features of senescent cells include cell cycle arrest, resistance to apoptosis, and a secretory phenotype known as the senescence-associated secretory phenotype (SASP), resulting in increased secretion of various bioactive factors (mainly cytokines, chemokines, proteinases, and growth factors) that have pro-inflammatory effects [20]. Prolonged SASP signaling can induce cytotoxicity or drive healthy cells into senescence and decline homeostatic immune function. Additionally, phagocytic cells that clear senescent cells can also become senescent, perpetuating inflammation and disease processes. Like other changes in cell fate, senescent cells exhibit abnormalities in their morphology, including large, flat bodies, and alterations in organelles, especially membranous ones, such as lysosomes, mitochondria, endoplasmic reticulum (ER), and plasma membrane, used as hallmark markers of senescence [21,22]. For example, the number and size of lysosomes are increased, and the lysosomal activity of the enzyme β-galactosidase (β-Gal) is used as a marker of cellular senescence [23].

A deregulated metabolic profile is linked to mitochondrial dysfunction, manifesting as respiratory defects that compromise the ability to generate ATP and contribute to elevated production of reactive oxygen species (ROS) [24]. Oxidative stress directly damages DNA, proteins, and lipids. Irreversibly misfolded proteins accumulate in the endoplasmic reticulum lumen, aggregate, and become toxic to cell survival. Adaptive mechanisms tend to restore the ER protein homeostasis, eliminating misfolded proteins, such as the ER-associated protein degradation (ERAD) system, the autophagy–lysosome pathway, and the unfolded protein response (UPR). Incompletely folded forms of proteins are eliminated by the ERAD system that dislocates them from the ER back into the cytosol where they are degraded by the ubiquitin–proteasome system [25,26]. When misfolded proteins accumulate above a critical threshold, cells activate the UPR, a dynamic signaling network that orchestrates the recovery of homeostasis or triggers apoptosis, depending on the level of damage [27,28,29]. In addition, the UPR controls other pathways of the lipid and energy metabolisms [30].

Despite the permanently arrested state of cell growth, senescent cells remain highly metabolically active [21,31]. A metabolic shift in substrate utilization from oxidative phosphorylation to glycolysis is required to cope with the energetic demands of the senescent program including secretion of pro-inflammatory SASP factors [32]. However, metabolic reprogramming also involves lipid species. Proteomic analysis revealed that senescent cells accumulate lipid droplets and display a signature of enhanced lipid metabolism, including proteins involved in lipid binding, biosynthesis, degradation, and storage [33].

**Figure 2:**
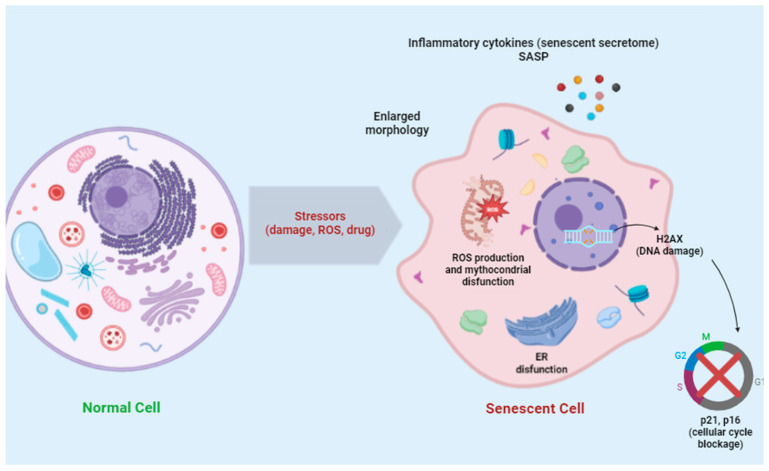
Hallmarks of cellular senescence and key features defining cellular senescence, including irreversible growth arrest, metabolic reprogramming, altered gene expression, secretion of molecules that trigger inflammation, and the senescence-associated secretory phenotype (SASP).

Various exogenous and endogenous stimuli may trigger the senescence process, including telomere shortening, mitogenic signals, excessive activation of oncogenes, accumulation of stress, metabolic disturbances, irradiation, and chemotherapeutic drugs. The diversity of stimuli leads to distinct yet interlinked senescence programs, each characterized by unique phenotypic changes [34] (Figure 2).

While cellular senescence is a systemic process, the mechanisms and consequences of senescence vary significantly across organs, reflecting distinct cellular compositions, regenerative capacities, and stress responses. For instance, in the lung, senescence contributes to impaired alveolar regeneration and persistent inflammation, promoting accumulation of fibroblasts secreting excessive amounts of extracellular matrix (ECM) components, central to diseases such as pulmonary fibrosis and chronic obstructive pulmonary disease [14]. In the pancreas, β-cell aging impairs β-cell function and reduces insulin sensitivity and secretion, thus predisposing T2DM development in the elderly senescence impairs insulin sensitivity and secretion, thus promoting metabolic dysfunction and predisposing the onset and progression of type 2 diabetes [2]. The crucial interplay between accumulation of senescent cells, inflammation, and fibrosis, also contributes to kidney dysfunction. These organ-specific manifestations of aging and senescence underscore the necessity of developing precise and non-invasive imaging strategies that can account for tissue heterogeneity.

This heterogeneity in senescence phenotypes across tissues has hindered the identification of a single, reliable biomarker for cellular senescence, posing a significant challenge for the development of targeted therapies (senotherapies). Not all senescent cells display all biomarkers of senescence. In addition, senescence biomarkers are not necessarily specific to senescent cells, increasing the risk of off-target effects from senotherapeutics, while some tissues may remain unaffected by therapeutics.

Recently, the first practical guidelines and recommendations called “Minimum Information for Cellular Senescence Experimentation in vivo” (MICSE) have been published for experimental research in mouse models aimed to identify the features and functions of senescent cells in vivo [35]. MICSE proposes a sequential approach starting with the demonstration of the cell cycle arrest, the most defining hallmark of senescence, through the detection of the cell cycle arrest proteins p21 and p16. As a second step, characterization of at least two auxiliary markers of cellular senescence is recommended, (i.e., LMNB1, HMGB1, SASP factors, SADS, ALISE, DNA damage, and β-gal). Finally, MICSE proposes the approaches to reduce and verify the activity of senescent cells by using senostatic or senolytic drugs. Senostatic drugs suppress the harmful secretions of senescent cells without killing them, reducing chronic inflammation and tissue damage. In contrast, senolytic drugs selectively eliminate senescent cells by targeting their survival pathways, thereby restoring tissue function and improving healthspan. Together, these approaches represent complementary strategies in aging and age-related disease therapy.

A deeper understanding of the organ-specific features of senescence as well as more precise biomarkers are required to improve the efficacy and safety of senotherapies.

Molecular imaging with positron emission tomography (PET) has emerged as a powerful tool for the non-invasive visualization and quantification of biological processes at the molecular level in vivo. Central to this technology are radioligands—molecules labeled with short-lived positron-emitting radionuclides such as Fluorine-18 (F-18), or Gallium-68 (Ga-68)—which serve as highly specific probes that bind to molecular targets of interest. These radioligands typically consist of a targeting moiety, designed to selectively bind biomarkers, conjugated to a radioactive isotope that emits positrons detectable by PET scanners. This combination enables precise localization, quantification, and dynamic tracking of target molecules within tissues and organs. The development of effective PET radioligands requires careful consideration of several factors, including target specificity, binding affinity, pharmacokinetics, metabolic stability, and safety. Once administered, radioligands circulate systemically, homing to their targets and providing real-time data on target expression, receptor density, enzyme activity, or cellular processes. Importantly, the short half-lives of commonly used radionuclides (e.g., F-18 ~110 min; Ga-68 ~68 min) balance sufficient imaging time with rapid clearance to minimize radiation exposure to patients. Due to their high sensitivity and ability to quantify targets at picomolar concentrations, PET radioligands are invaluable in both research and clinical settings. They enable longitudinal monitoring of disease progression, therapeutic target engagement, and treatment response across a range of conditions such as cancer, neurodegenerative disorders, metabolic diseases, and inflammatory states.

Over the past decade, significant advancements have been made in developing novel PET radioligands targeting pathways implicated in cellular senescence. These include probes directed at lysosomal enzymes such as β-Gal, chemokine receptors, and components of the extracellular matrix, which are key hallmarks of senescent cells. This expanding toolkit is shifting molecular imaging from descriptive to highly quantitative and target-specific applications. Such progress opens new avenues for earlier diagnosis, precise monitoring of senolytic and senostatic therapies, and ultimately, personalized management of age-related diseases driven by senescence.

This narrative review highlights the potential of PET radioligands, starting with our experience in developing and validating them, followed by imaging the hallmarks of senescence, and finally, their translation into clinical interventions.

## 2. The PET Imaging Approach for In Vivo Detection of Senescence Processes

The most widely used radiotracer for PET imaging is ^18^F-fluorodeoxyglucose ([^18^F]FDG), a glucose analog which measures glucose consumption. Like glucose, [^18^F]FDG enters into living cells through glucose transporters (GLUTs). Unlike glucose, when phosphorylated by hexokinases, it is intracellularly trapped as FDG-6-phosphate.

Increased glycolysis has been repeatedly associated with senescent cells in culture [36,37]. Activation of glycolysis occurring in senescent immune cells affects macrophage polarization and inflammatory response. Senescent macrophages tend to polarize towards the M1 phenotype and exhibit glycolytic metabolism [38]. GLUT1 is highly expressed in senescent cells [39] and aged lungs and regulates fibrogenesis [40]. At an old age, reduced neuroplasticity and loss of synaptic function may be reflected by a reduction in neuronal [^18^F]FDG uptake [41], and FDG-PET is a well-established tool in measuring changes in brain glucose metabolism in both normal and pathological aging [42,43].

The aging process induces physiological alterations in body composition, including decline in muscle mass, reduction in bone density, and increase in fat mass, which can negatively impact a person’s well-being and healthy aging. FDG-PET can quantify glucose metabolism of the various tissues. Moreover, combination with computed tomography (CT) in the current hybrid PET/CT systems provides information on bone density, quantity, and the distribution of adipose tissue and skeletal muscle mass. It has been recently demonstrated that changes in body composition during aging differ significantly by sex and that glucose metabolism is closely linked to fat quantity, distribution, and metabolic conditions [44]. FDG-PET/CT enables the characterization of distinct patterns of age-related changes in body composition and glucose metabolism, with significant differences between sexes.

Aging is also one of the most important determinants of the functional status of brown adipose tissue (BAT), and BAT activity is closely related to obesity, type 2 diabetes, and atherosclerosis [45,46]. FDG-PET imaging provides in vivo direct measurement of BAT functional status [47] which can be directly calculated from the glucose metabolism activity levels [48], that is, the intensity of the PET signal, a function of BAT activation [49].

In the aging heart, molecular imaging by means of FDG-PET demonstrated the association between metabolic syndrome and changes in cardiac metabolism [50]. In healthy individuals, absence of cardiac [^18^F]FDG uptake resulted in higher insulin resistance index (HOMA-IR), and higher prevalence of hypertension, diabetes, and early atherosclerosis. During follow up, recovery of myocardial [^18^F]FDG uptake was associated with the improvement in cardiometabolic profile.

In addition to identifying synaptic vulnerability using FDG-PET, development in the radiopharmaceutical field provides tracers with the ability to target brain changes associated with the aging process [51], such as beta-amyloid deposition and tau inclusions [52,53], alteration in neurotransmitter dynamics [54,55], and neuroinflammation [56]. Cellular senescence can in fact trigger neurodegeneration via multiple mechanisms, including metabolic dysregulation, protein unfolding and aggregation, and microglial activation [57].

Finally, extraction of quantifiable features from PET images, as surrogates of the rates of biological processes, allow early diagnosis and assessment of treatment response, and hence, improving sensitivity in identifying patients who do not benefit from treatment. A clear cut-off derived from databases of healthy controls provides a precise characterization of neurodegeneration phenotypes and allows accurate selection for clinical trials [58].

**Figure 3:**
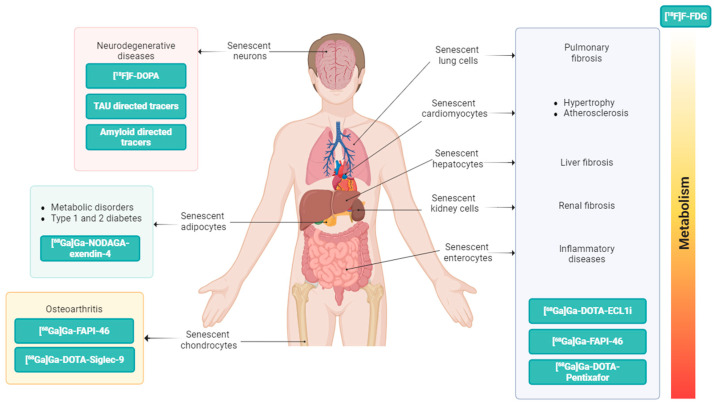
PET imaging probes based on senescence-related biomarkers that are able to reveal in vivo changes associated with aging.

However, a more selective targeting of the senescence process is needed to address this complex and multifaceted process that is directly involved as a key driver of aging and age-related diseases. Technologies allowing the targeting of senescence-associated pathways in organs and tissues may enable the development of a more precise strategy for eliminating senescent cells, preventing cellular senescence, developing new treatments, including choice of senotherapeutic or senolytic drugs, route of administration, and scheduling protocols. In recent years, along-side the identification of senescence-associated biomarkers, including β-Gal, G1/S cell cycle checkpoint inhibitors (like p16Ink4a, p21CIP1, p53), SASP factors (such as chemokines, IL-6, IL-8, MMPs), mTOR and AMPK pathways, and sirtuin signaling [59], more specific strategies that are able to precisely refine the senescent cells phenotype both in vitro and in vivo have been developed [60]. Recently, several targeted radioligands have emerged as translational tools able to reveal distinct features of the senescence process (Table 1 and Table 2), and some age-related biomarkers have been developed into imaging probes for PET, used to characterize age-related changes in vivo (Figure 3).

### 2.1. PET Tracers Targeting Lysosomal β-Galactosidase (β-Gal)

As previously reported, senescent cells undergo significant changes in both morphology and metabolism, with especially notable alterations in their lysosomes [21,22]. Upregulation of many lysosomal proteins and increased lysosomal content is accompanied by elevated activity of the lysosomal enzyme β-galactosidase (β-Gal), also known as senescence-associated β-galactosidase (SA-β-Gal) [61,62]. Its existence was first proposed by Dimri et al. in 1995 [61] based on the observation that only senescent cells develop staining with a β-Gal assay at pH 6.0. In addition, the activation of β-Gal is associated with various stressors, such as DNA damage, oxidative stress, and telomere shortening.

For this reason, β-Gal is one of the most commonly used biomarkers for senescence and has been effectively utilized in fluorescent-based small-molecule probes to visualize senescent cells at both cellular and organismal levels [63]. Unfortunately, most of these probes are unsuitable for in vivo imaging due to their short fluorescence emission wavelengths, which limit tissue penetration. To overcome this limitation, over the years, senescence probes compatible with clinical imaging modalities, such as PET, have been developed [64].

In 2018, a ^18^F-radiolabeled probe targeting β-Gal, called [^18^F]FPyGal, was patented for PET imaging of cellular senescence in the development of osteoarthritis [65]. The inventors demonstrated in in vitro (primary chondrocytes) and then in vivo (small animal model-mice and a large animal model-pig) that [^18^F]FPyGal could selectively accumulate in senescent cells, as β-gal activity would cleave the probe, trapping and enriching the ^18^F signal within those cells, thereby enabling the detection of cellular senescence via PET [65]. Currently, a Phase I/II clinical trial (NCT04536454) is ongoing to evaluate [^18^F]FPyGal safety, radiation exposure, and diagnostic accuracy for tumor senescence imaging in cancer patients with rectum cancer, non-small cell lung cancer, and esophagogastric junction adenocarcinoma undergoing neoadjuvant therapy, with an enrollment of 32 oncological patients. The relationship between histopathological senescence markers (p16, p21, p53) and [^18^F]FPyGal signals is also being investigated in surgically resected samples post-therapy, compared to treatment-naive biopsy samples.

Recently, another PET imaging probe, [^68^Ga]Ga-βGal, was synthesized, with a high labeling yield (90 ± 4.3%) and radiochemical purity (>95%), to visualize β-Gal activity in living mice with genetically edited tumors and in chemotherapy-induced senescence models [64]. The probe successfully detected cellular senescence both in vitro and in vivo. In mice, PET imaging revealed significant uptake (3.13 ± 0.75%ID/g) and retention of the probe in CT26.CL25 tumors with β-Gal activity following intravenous injection, in contrast to the controls. With its unlimited penetration depth and exceptional sensitivity, this PET tracer has enabled quantitative, whole-body detection of cellular senescence in diverse contexts. Although in most studies the use of these probes is limited to the oncologic field, the application of radiolabeled β-Gal could be extended to other diseases.

### 2.2. PET Tracers Targeting Lipofuscin

Lipofuscin is a type of membrane-bound cellular waste that cannot be degraded or expelled from the cell; it can only be diluted through cell division and subsequent growth. Postmitotic cells, like neurons and cardiomyocytes, inevitably accumulate lipofuscin, which is recognized as a reliable biomarker for cellular age [66]. In the aging human brain, lipofuscin deposits are not evenly distributed but are concentrated in specific regions of interest [67,68]. Although the exact mechanism behind lipofuscin formation remains unclear, it is believed that the primary source of lipofuscin arises from incomplete lysosomal degradation of damaged mitochondria and ROS produced during senescence. Elevated ROS levels can lead to the carbonylation of various protein residues, including proline, threonine, lysine, and arginine, in the presence of metals. These carbonyl residues can then react with amino groups to form Schiff bases, promoting protein aggregation. Ultimately, insoluble lipofuscin is formed through the cross-linking of these aggregates with sugars and lipids [69]. Lipofuscin cytotoxicity is partly due to its ability to incorporate transition metals, such as iron and copper, forming a redox-active surface. Intracellular lipofuscin disrupts essential cellular processes like the ubiquitin–proteasome pathway (UPP) and the autophagy–lysosomal pathway, both vital for clearing damaged organelles and oxidized proteins. Impairment of these pathways can lead to increased lipofuscin accumulation, reduced cell viability, resulting in what is referred to as a “garbage catastrophe” [67]. Significant lipofuscin buildup has been observed in several neurodegenerative diseases, such as Alzheimer’s and Parkinson’s diseases. Additionally, higher levels of lipofuscin accumulation have been reported in the later stages of heart failure, including dilated cardiomyopathy and ischemic cardiomyopathy [66].

Initially, Georgakopoulou et al. (2013) demonstrated that senescent cells can be specifically identified using the histopathology dye Sudan Black B (SBB) [68], which stains lipofuscin granules. To enhance the sensitivity and specificity of this staining method, a biotin-linked derivative of SBB for antibody-enhanced detection of lipofuscin was created [70].

Although the cytotoxicity of lipofuscin has been documented in various pathological conditions, promoting a senescent phenotype to suppress tumor growth could be a strategy for the development of new anticancer therapies and PET tracers to assess treatment response. To this end, Brickute et al. synthesized the first radiolabeled derivative of SBB to take advantage of the exceptional sensitivity of PET in detecting and quantifying lipofuscin in oncogene-induced senescence (OIS) and therapy-induced senescence (TIS) [71]. The targeted radiolabeled molecule, [^18^F]FET-SBB, was synthesized using the [^18^F]FEA prosthetic group, achieving a 5% radiochemical yield (RCY), uncorrected for decay, within 120 min. The molar activity (Am) was 6.9 GBq μmol^−1^, with radiochemical purity (RCP) exceeding 95% [62]. The log D7.5 determination value of 1.57 ± 0.18 suggested that the compound is highly lipophilic. After the establishment of the in vitro senescent model, i.e., MCF-7 (breast carcinoma), T47D (breast carcinoma), and HCT116 (colorectal carcinoma), tracer uptake was assessed. Tracer retention in T47D cells increased significantly with antineoplastic treatment (palbociclib), while [^18^F]FET-SBB uptake decreased in MCF-7 cells. This reduction may result from palbociclib-induced upregulation of efflux transporters, which can rapidly expel the tracer. Although direct evidence is lacking, palbociclib is known to be a substrate for efflux proteins like P-gp and BCRP, highlighting a potential limitation of radioactive imaging agents due to ATP-binding cassette transporter activity. Then, the pharmacokinetic profile of [^18^F]FET-SBB in vivo using PET imaging and radioactive metabolite analysis was evaluated, revealing substantial liver accumulation and predominantly intact parent radiotracer at 60 min post-injection. Altogether, these results show that the in vitro uptake of [^18^F]FET-SBB in a senescent cell line correlated with lipofuscin deposits; in vivo PET imaging and metabolite analysis confirmed a favorable pharmacokinetic and metabolic profile, suggesting a need for further studies in animal models with senescent tissues.

### 2.3. PET Tracers Targeting Chemokines

A persistent, low-grade inflammation has emerged as an important driving contributor to age-related diseases. Senescent cells secrete pro-inflammatory mediators stimulating the accumulation of ROS, further accelerating the deterioration process [72]. As these senescent cells accumulate, they perpetuate a cycle of inflammation that exacerbates tissue degeneration and contributes to various age-related conditions. Chemokines, particularly chemokine ligand 2 (CCL2) and chemokine ligand 12 (CXCL12), play a crucial role in influencing the aging process, by regulating primarily through inflammatory responses (inflammaging), recruitment of macrophages, and orchestrated trafficking of other immune cells.

CCL2, which is secreted by a wide range of cells, including macrophages, smooth muscle cells, and endothelial cells, can trigger a cascade of intracellular signaling events upon binding to its receptor, CCR2. This signaling pathway, which involves PI3K/AKT, p38 MAPK, and JAK/STAT3, leads to cellular processes such as proliferation, migration, and inflammation, which are essential for leukocyte chemotaxis and tissue remodeling. It has been shown that circulating levels of CCL2 are increased in aged individuals [73] and that aging increases the frequency of pro-inflammatory CCR2+ macrophages in the heart and the kidney [74,75]. The activation of pro-inflammatory macrophages can exacerbate cardiac pathology and disease progression [76]. In a progeroid animal model, CCL2 overexpression promoted the death of the mice, accelerated the development of degenerative changes, and caused metabolic dysregulation [77]. Inflammation and ectopic fat deposition in the aging murine liver is influenced by CCR2 [78], and the upregulation of CCL2 expression has been associated with cellular senescence in obese adipose tissue contributing to chronic inflammation [79]. Recruitment of CCR2-positive macrophages promotes renal injury and fibrotic remodeling in glomerulonephritis and diabetic nephropathy. Moreover, increased glomerular expression of CCR2 and CCL2 has been demonstrated in human focal glomerulosclerosis [80].

The CCL2/CCR2 axis, therefore, plays a significant role in promoting the chronic inflammation observed in aging, contributing to the progression of age-related diseases, such as diabetes, cardiovascular lesions, tumors, and complications related to atherosclerosis, including stroke, myocardial infarction, and heart failure.

Similarly, in the brain, the CCL2-CCR2 axis plays a role in neuroinflammation, contributing to neurodegeneration and cognitive decline. In animal models, CCL2 overexpression has been shown to promote cognitive impairment and accelerate β-amyloid deposition in Alzheimer’s disease. Human studies also suggest that higher CCR2 expression is associated with poorer cognitive function, underscoring its involvement in age-related cognitive decline. This pathway has also been implicated in the pathogenesis of obesity-related chronic inflammation and cellular senescence in adipose tissue, further linking CCL2/CCR2 signaling to systemic inflammation seen in aging. Thus, the CCL2/CCR2 axis represents a pivotal mechanism in aging, with its dysregulation contributing to the progression of chronic inflammation and tissue damage across multiple organ systems. Targeting this pathway may offer diagnostic and therapeutic potential for mitigating the impacts of inflammaging and improving health outcomes in the elderly.

PET imaging probes targeting CCR2 expression have been developed and radiolabeled with Ga-68 or Cu-64, using the extracellular loop 1 inverso (ECL1i) peptide as a vehicle [81,82] that binds to the extracellular domain of CCR2. The tracer [^64^Cu]Cu-DOTA-ECL1i, enabled detection of CCR2+ monocyte and macrophage accumulation in the lung following ischemia–reperfusion injury or lipopolysaccharide challenge; however, its use is limited by the need for a cyclotron to produce Cu-64, the radionuclide’s long half-life (12.7 h), and associated radiation concerns that hinder serial imaging in acute settings [82,83]. To address this, recently, a fully automated process has been used to produce [^68^Ga]Ga-DOTA-ECL1i, investigating different amounts of precursor ligand (10 – 20 – 30 – 40 - 50μg). The evaluated parameters to test [^68^Ga]Ga-DOTA-ECL1i safety reported that a radiochemical purity of 100%, radiochemical yield of 66.69%, and good molar activity of 45.41 GBq/μM were achieved using 20 μg of peptide precursor [81]. Moreover, the radioligand pH value remained unchanged (pH = 7) over 3 h. In a few studies, [^68^Ga]Ga-DOTA-ECL1i has been used to image the recruitment of inflammatory CCR2+ monocytes/macrophages in the mouse model of injured heart [83] and in human ST-segment elevation myocardial infarction [84]. Autoradiography studies confirmed radiolabeled-DOTA-ECL1i binding to human heart sections of patients with heart failure [83]. Compared with the [^64^Cu]Cu-DOTA-ECL1i probe, the [^68^Ga]Ga-DOTA-ECL1i tracer provides distinct advantages that expand the scope of CCR2 imaging. Its shorter half-life reduces radiation burden, while the positive charge promotes faster clearance. The availability of ^68^Ge/^68^Ga generators enables convenient on-site production, supporting multiple dosing and serial imaging in acute settings. In addition, its low hepatic retention enhances suitability for liver studies, broadening its potential clinical applications [83]. These findings establish the feasibility of molecular imaging of CCR2 expression to identify and quantify the extent of inflammation after myocardial injury [85].

Another specific radioligand, [^68^Ga]Ga-DOTA-Pentixafor, has been recently developed to detect the C-X-C motif chemokine receptor 4 (CXCR4) [86], a G-protein-coupled seven-span transmembrane receptor protein, which plays a pivotal role in the recruitment of immune cells to injured and inflamed tissue. CXCR4 is activated by the chemokine CXCL12, also known as stromal-derived factor (SDF)-1, and its role has been demonstrated in tumor development [87], HIV-1 infection [88], and acute myeloid leukemia [89]. Analogous to the CCL2/CCR2 axis, the CXCL12/CXCR4 signaling pathway is also critically involved in a range of aging-related diseases, including cardiovascular pathologies, neurodegenerative disorders, and metabolic dysfunctions. For instance, the chemokine receptor CXCR4 is significantly upregulated after kidney injury, and its sustaining activation aggravates the fibrotic responses [90,91,92]. Furthermore, it is overexpressed not only in pulmonary fibrosis but also in other fibrotic interstitial lung diseases (ILDs) [93], particularly within honeycomb cysts and distal airway epithelium [94].

CXCR4-based PET imaging may be used as a diagnostic tool for disease activity assessment and prognostication in ILDs [95], lung fibrosis [96], atherosclerosis [97] or rheumatoid arthritis [98]. A recent study demonstrated that Pentixafor could be radiolabeled with Ga-68 using a fully automated synthesis, yielding three consecutive batches with radiochemical purities of 99.86%, 99.83%, and 100%, and radiochemical yields of 56.7%, 56.9%, and 57.3%, respectively, representing an improvement from previously reported results [86]. The role of [^68^Ga]Ga-DOTA-Pentixafor PET scan is to detect leukocyte infiltration, and recruitment has been highlighted in animal and human studies where it showed CXCR4 upregulation in the highest fibrotic areas with data in line with the immunohistochemistry analysis [96].

Developing CXCR4 antagonists has become a novel strategy for anti-inflammatory therapy [99,100]. A dysregulation of CXCR4 expression has been demonstrated in brains of subjects with progressive supranuclear palsy (PSP), frontotemporal dementia (FTD) and Parkinson’s disease (PD), and in mouse models of tau pathology [101]. CXCR4 is highly expressed in the microglia and astrocytes which are central mediators of inflammatory responses in the central nervous system. The activation of CXCL12/CXCR4 axis might be involved in the pathological dysfunction of AD contributing to neurotoxic stress within the neuro-inflammatory cascade though the secretion of pro-inflammatory factors [102] and the release of glutamate from astrocytes, impacting neuronal function and apoptosis. Pharmacological blockade of CXCR4/CXCL12 interactions has been shown to reduce neuronal apoptosis, significantly alleviating Alzheimer’s disease (AD)-associated pathologies and cognitive deficits [103].

The wide spectrum of functional involvements of chemokines in aging and associated diseases is the base of several trials aiming to improve lifespan and promote healthy aging [104]. The development of PET probes targeting the chemokine receptors allows the assessment of their engagement in disease and investigate drug–receptor interactions and provides insights into the pathophysiology of various diseases, including cardiovascular and pulmonary disorders.

### 2.4. PET Tracers Targeting Siglecs Family

**Figure 4:**
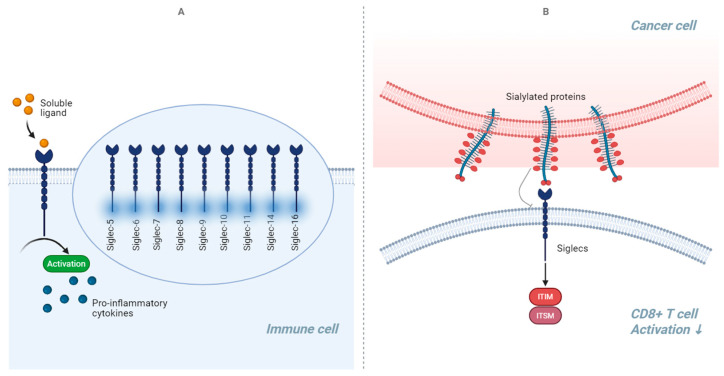
The sialic acid-binding immunoglobulin-like lectins (Siglecs) function as transmembrane receptors binding to sialic acid residues on cell-surface glycans. Siglecs are involved in immune signaling as immune checkpoints able to regulate immune responses in inflammatory diseases (**A**) as well as in cancer (**B**). In inflammatory conditions, engagement of inhibitory Siglecs dampens immune cell activation, curbing excessive inflammation but sometimes impairing pathogen clearance. Conversely, in cancer, tumor cells exploit this pathway by upregulating sialic acid ligands, engaging Siglecs on immune cells to suppress anti-tumor responses (blue arrow indicates a decrease in T-cell activation) and promote immune evasion.

Recent studies have underscored the role of the sialic acid-binding immunoglobulin-like lectin (Siglec) family of receptors in regulating cellular senescence, highlighting their connections to immune responses, inflammation, and cellular aging [105]. Siglecs belong to the immunoglobulin superfamily and primarily recognize sialic acid residues on glycoproteins and glycolipids. These receptors are mainly expressed on immune cells, where they play crucial roles in modulating immune responses, self-tolerance, and inflammation (Figure 4) [106]. In the central nervous system (CNS), Siglecs can exhibit both neuroprotective and neurotoxic effects, depending on the specific context and the Siglec involved, thereby influencing the senescence process in various ways [105]. For instance, Siglec-7 and Siglec-9 have been shown to modulate the secretory phenotype of senescent cells by interacting with sialylated ligands, helping to mitigate inflammatory responses arising from these cells and reducing the harmful effects of the senescence-associated secretory phenotype on surrounding tissues [107]. Conversely, Siglecs can facilitate immune evasion in senescent cells by promoting the recognition of sialylated self-antigens, which may lead to altered immune responses. This can result in senescent cells persisting longer than necessary, potentially contributing to tissue dysfunction and age-related pathologies. Additionally, in the brain, microglia—typically protective—can exacerbate inflammatory responses and exhibit neurotoxic effects under certain conditions due to the expression of various Siglecs, including CD33 (Siglec-3), Siglec-11, and Siglec-16 in humans [107,108].

Recent advances in molecular imaging have focused on the development of radiolabeled peptides targeting Siglec-9 for the non-invasive detection of inflammation and related pathologies. Currently, the peptide Siglec-9 has been successfully labeled with Ga-68 to visualize vascular adhesion protein-1 (VAP-1) in sites of inflammation, cancer, and rheumatoid arthritis [109,110,111]. This radiolabeling approach yielded a non-decay-corrected radiochemical yield of 62%, with a radiochemical purity exceeding 98% and a specific radioactivity of 35 MBq/nmol [112]. In a first-in-human study evaluating the potential of [^68^Ga]Ga-DOTA-Siglec-9 for imaging inflammation, the radiotracer was administered intravenously and found to be safe and well-tolerated in a cohort of six subjects. [^68^Ga]Ga-DOTA-Siglec-9 exhibited rapid clearance from the bloodstream, with several radioactive metabolites identified. The highest absorbed radiation doses were observed in the urinary bladder wall (0.38 mSv/MBq) and kidneys (0.054 mSv/MBq), with a mean effective dose of 0.022 mSv/MBq (range: 0.020–0.024 mSv/MBq). Notably, [^68^Ga]Ga-DOTA-Siglec-9 demonstrated comparable performance to [^18^F]FDG in the detection of arthritis [111]. In addition to Ga-68, Siglec-9 has also been labeled with F-18, resulting in compounds such as [^18^F]FDR-Siglec-9, which were produced with high radiochemical quality and sufficient specific radioactivity to enable effective in vivo PET imaging of experimental inflammation [109,110,111,112,113]. These developments underscore the potential of Siglec-targeted radiopharmaceuticals not only for diagnostic imaging but also for therapeutic strategies aimed at modulating the senescence process, reducing chronic inflammation, and promoting tissue regeneration.

### 2.5. PET Tracers Targeting GLP-1 Receptor

Glucagon-like peptide-1 receptor (GLP-1R) is a key regulator of glucose and energy metabolism, emerging in recent years as a prominent pharmacological target for type 2 diabetes mellitus (T2DM) and obesity. GLP-1R is a G protein-coupled receptor with a large extracellular domain and a seven-times-transmembrane core domain with α-helix bundles. In addition to pancreatic ꞵ-cells, it is localized in the gastrointestinal tract but also detected at lower levels in the central nervous system, the heart, vascular smooth muscle, and endothelial cells [114]. Glucagon-like peptide 1 (GLP-1) serves as the primary agonist for GLP-1R in vivo and activates the receptor. Mechanistically, this binding lowers blood glucose levels without increasing hypoglycemia risk, triggering the activation of G-proteins and leading to an increase in the intracellular second messenger cAMP, followed by the activation of protein kinase A (PKA) and the indirect activation of epidermal growth factor receptor, followed by phosphoinositide 3-kinase (PI3K) and Akt signaling, crucial for the survival and function of pancreatic β-cells [115,116]. Activation of GLP-1R by GLP-1 has been found to regulate several inflammatory pathways, including cytokine production, oxidative stress, and the recruitment of immune cells [117]. The generated persistent and chronic inflammation is a risk factor for atherosclerosis, diabetes, cardiovascular disease, neurodegenerative disorders, nonalcoholic steatohepatitis, and nephropathy. For instance, in both diabetes and atherosclerosis, endothelial dysfunction is directly linked to disease progression [118,119]. A major factor driving this process is the elevated production of ROS, which causes increased intracellular damage, including DNA damage, potentially triggering apoptosis or cellular senescence. Elevated levels of cellular senescence have been identified in the blood vessels of patients with coronary artery disease, as well as in the tubular regions of those with diabetic nephropathy [118,119].

Considering the interconnected role of GLP-1R, there is a growing interest in investigating the receptor involvement in age-related pathologies. As evidenced, aging is a main risk factor for the development of T2DM, as the β-cell mass diminishes with age. Chronic insulin exposure, occurring in T2DM, has been demonstrated to induce senescence in pancreatic β-cells, hepatocytes, and adipose tissue [10,120]. In animal studies, elimination of senescent cells improves blood glucose levels and reduces diabetic complications [121].

In recent years, several GLP-1R agonists (GLP-1RAs), mimicking the action of endogenous GLP-1, have been successfully developed for T2DM treatment. GLP-1RAs are structurally modified versions of GLP-1 designed to resist degradation by the enzyme dipeptydil-peptidase 4 (DPP-4), which extends their half-life and enhances their biological effects. Also, these novel drugs have demonstrated effectiveness beyond metabolic diseases, with beneficial activities in the brain [122], heart [123,124], and lung [125], sustained notably by their strong anti-inflammatory activities [114,126]. Recent evidence has revealed that GLP-1RAs exert anti-inflammatory effects by downregulating NF-κB signaling and reducing SASP factors, thus potentially alleviating senescence-related tissue dysfunction [118]. Moreover, GLP-1RAs reduce macrophage infiltration in blood vessels and the production of pro-inflammatory mediators such as IL-6, IL-1β, TNF-α, and CRP [117]. The anti-inflammatory effects of GLP-1 agonists have been demonstrated in obstructive pulmonary disease in female C57BL/6 mice [127] and in a mouse model of Alzheimer’s diseases, where the treatment with GLP-1RAs reduced the inflammatory response in the cortex by decreasing the activated microglia [128]. In a murine model of AD, administration of GLP-1RAs reduced the levels of pathological markers associated with AD, including amyloid plaque load [129]. In osteoarthritis, inflammation is closely related to the activation of macrophages [130], which express GLP-1R. Macrophages accumulate in the synovial membrane and subchondral bone, release pro-inflammatory cytokines, and determine the inflammatory response and the degradation of joint cartilage. In the treatment of Parkinson’s and Alzheimer’s diseases, GLP-1RAs have shown a neuroprotective potential in slowing disease progression. For example, liraglutide prevents a decrease in cerebral metabolic rate for glucose in AD patients compared with placebo [131], while long-term treatment with dulaglutide demonstrated beneficial effects on cognitive impairment in patients aged ≥50 years with T2DM [132], and a 12 month-exenatide treatment significantly improved motor and cognitive functions in PD patients [133]. In the coronary atherosclerotic process, GLP-1RAs may offer a protective effect by modulating macrophage polarization towards the M2 type [134].

More recently, the application of GLP-1RAs in the field of nonalcoholic fatty liver disease (NAFLD) has gained widespread attention [135]. NAFLD is an umbrella term to describe the spectrum of nonalcoholic fatty liver disease, including hepatic steatosis. NAFLD can evolve toward the more severe nonalcoholic steatohepatitis or NASH, a state of macrovesicular steatosis and hepatic inflammation, with or without fibrosis. GLP-1RAs have been demonstrated to significantly improve the clinical, biochemical, and histological markers of hepatic steatosis, inflammation, and fibrosis in animal models and in patients with NAFLD [136,137].

**Figure 5:**
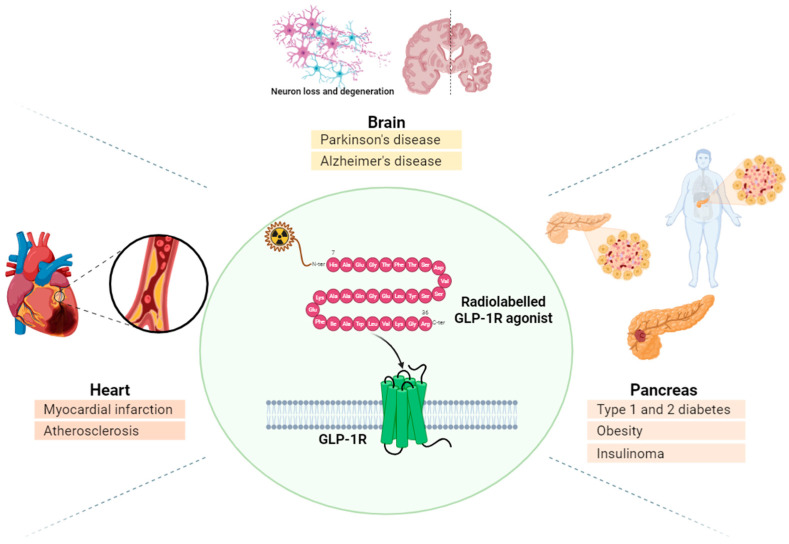
GLP-1 receptor-based targeted drugs have been radiolabeled with either gamma emitters or positron emitters to non-invasively monitor GLP-1 expression in several organs.

Many GLP-1R-based targeted drugs are currently evaluated in clinical studies for the development of next-generation diabetes drugs, due to their potential in the treatment of various aging-related diseases. In this context, in vivo visualization and quantification of GLP-1R expression using PET with selective radiotracers assume great importance from both a diagnostic and therapeutic point of view (Figure 5). Recently, the GLP-1 agonist Exendin-4 has been radiolabeled with either gamma emitters (i.e., Iodium-123, Technitium-99m) [138] or positron emitters (i.e., Ga-68, F-18) to image pancreatic tumors (i.e., insulinoma) and to assess safely and non-invasively pancreatic β-cell density [139]. Few groups validated the radiosynthesis of [^68^Ga]Ga-NODAGA-Exendin-4 with high specific radioactivity, high binding affinity to GLP-1R, relative stability in vivo, and rapid clearance from the blood [140,141,142]. Exendin-4 PET/CT has been used for non-invasive quantification of β-cell mass in T1DM [143] for monitoring β-cell survival after intrahepatic islet transplantation in T1DM [144] or to study the development of T2DM [145]. Carlsson et al., at Uppsala University, (Sweden), have recently used [^68^Ga]Ga-NODAGA-Exendin-4 to monitor the first-in-human transplantation of genetically modified allogeneic donor islet cells into a man with long-standing T1DM [146]. GLP-1R-based PET signal was localized at the injection sites in the patient’s forearm muscle and persisted 12 weeks after transplantation with no immunosuppression.

In another study, [^68^Ga]Ga-NODAGA-Exendin-4 PET/CT was able to detect increased myocardial GLP-1R expression in rats following myocardial infarction (MI) [147]. Tracer uptake was significantly elevated in the infarct region one week post-MI and correlated with the presence of CD68-positive macrophages and α-smooth muscle actin (α-SMA)-positive interstitial cells, suggesting an association with myocardial repair processes. Together, the presence of α-SMA and CD68-positive macrophages is often used as a marker of myofibroblast activation and to quantify macrophage infiltration and inflammation in tissue samples, respectively. These findings would support the use of Exendin-PET as a non-invasive method to monitor GLP-1R expression during healing and ventricular remodeling, and potentially evaluate myocardial repair mechanisms post-infarction.

Age-related changes in GLP-1R expression have been studied in aged rats using PET with [^18^F]AlF-NOTA-MAL-Cys^39^-Exendin-4 [148]. Significant reduction in GLP-1R expression was found in the olfactory, striatum, hypothalamus, substantial nigra, and hippocampus, and imaging data were confirmed by immunohistochemistry.

Overall, PET imaging with a specific radioligand targeting GLP1-R provides non-invasive assessment of receptor expression. High sensitivity of the PET technique allows the quantification of longitudinal changes in GLP-1R in organs, as well as the evaluation of target engagement of novel GLP-1 agonists. In vivo imaging of GLP-1R modulation in tissues could contribute to a deeper understanding of senescence processes correlated to hyperglycemia- and obesity-related metabolic changes, pathophysiology of hyperglycemia effects, and T2DM pathophysiology, as well as allow a personalized approach to disease management.

### 2.6. PET Tracers Targeting Fibroblast Activation Protein

**Figure 6:**
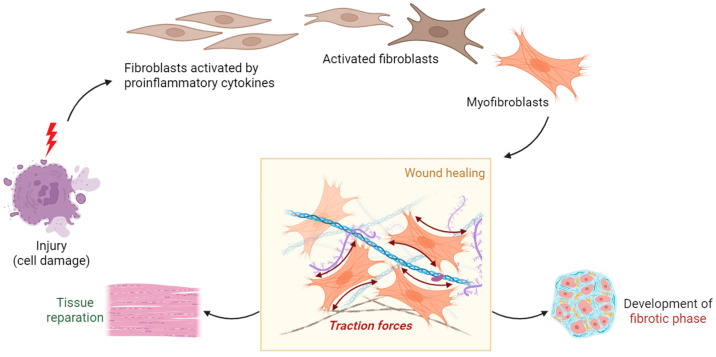
After tissue injury, fibroblasts transform into activated myofibroblasts, a key step in wound healing and fibrosis. This process is marked by a strong upregulation of fibroblast activation protein (FAP), especially during tissue remodeling.

Fibroblast activation protein-α (FAP) is a type-II transmembrane serine protease almost exclusively upregulated on activated fibroblasts or cancer-associated fibroblasts (CAFs). SASP can stimulate a fibrotic phenotype in fibroblasts through various pro-inflammatory mediators such as TGF-β1, IL-1β, IL-6, and PDGF, and other growth factors [149]. Upon inflammatory stimuli, fibroblasts are activated, leading to their differentiation into myofibroblasts—contractile cells that express α-SMA and deposit large amounts of extracellular matrix (ECM) participating in the fibrotic process [14]. This transition to myofibroblasts is intimately associated with the upregulation of FAP [150]. Senescent parenchymal cells (such as alveolar epithelial cells, renal tubular epithelial cells, and hepatocytes) release ROS and profibrotic factors, changing the local microenvironment and inducing the activation of adjacent interstitial cells (such as fibroblasts and stellate cells), with a fibrotic feedforward circuit (Figure 6) [151]. Activated fibroblasts are extensively involved in many age-related disorders, including cancer, fibrosis, arthritis, atherosclerosis, autoimmune diseases, and metabolic diseases.

In most solid tumors, FAP overexpression on CAFs is crucial in constructing and maintaining the tumor microenvironment (TME) [152]. FAP overexpression in CAFs correlates with tumor development, invasiveness, and progression [153]. For its specific and relatively stable expression in the tumor microenvironment, FAP has recently gained attention as a potential new target for CAR-T cell therapy [154] and various FAP vaccines in the preclinical and clinical setting [155].

In other pathological conditions, for instance, in idiopathic pulmonary fibrosis (IPF), FAP is specifically upregulated in fibroblastic foci and in the fibroblastic interstitium but not in adjacent normal tissue or in lung tissue from healthy individuals [156], and levels of FAP expression in the lungs correlate to the severity of IPF [157]. In fibrotic tissue, senescent cells are abundant and their elimination significantly improved lung health in the mouse models of pulmonary fibrosis [158]. In liver fibrosis, FAP is expressed in a subset of activated fibroblasts and hepatic stellate cells (HSCs), promoting macrophage infiltration and HSC profibrogenic activity [159]. In the mouse model of CCl4-induced fibrosis, pharmacological inhibition of FAP modulates macrophage function, attenuates parenchymal liver fibrosis, and promotes liver regeneration [159]. In musculoskeletal diseases, FAP has been identified as a critical player in the destruction of bone and cartilage tissues in rheumatoid arthritis and osteoarthritis, sustaining inflammation through the production of chemokines and cytokines and mediating joint damage. In mouse models of arthritis, deletion of FAP+ fibroblasts suppressed both inflammation and bone erosions [160].

Fibrosis is observed in nearly every form of myocardial disease. Following myocardial injury, fibroblasts undergo activation and myofibroblast transdifferentiation. Activated fibroblasts are critically implicated in the repair and remodeling of the injured myocardium to preserve its structural integrity. Excessive or prolonged activation may reduce ventricular compliance and promote adverse remodeling and can lead to the development of heart failure, arrhythmia, and major adverse cardiovascular events. Recently, an mRNA-based FAP-targeted CAR-T encapsulated in lipid nanoparticles (LNPs) has been developed to treat cardiac diseases. Applied in a mouse model of heart failure, this FAP-targeted CAR-T cell approach resulted in a notable reduction in cardiac fibrosis [161].

Altogether, FAP represents a crucial molecular link between fibroblast activation, ECM remodeling, and senescence. Its selective expression in pathological conditions and versatile functions in matrix regulation make FAP an ideal target for imaging and therapy. Recent advances in molecular imaging have leveraged FAP’s unique expression by developing radiolabeled peptides for FAP-based PET imaging. Several quinoline-based FAP inhibitors (FAPIs), notably FAPI-04 and FAPI-46 [162,163], have been developed, coupled to the chelator DOTA, enabling incorporation of ^68^Ga [164,165]. Given that FAP expression precedes fibrogenesis, both [^68^Ga]Ga-FAPI-04 and [^68^Ga]Ga-FAPI-46 have been evaluated for the detection of the fibrotic activity and monitoring disease progression in animal model [166] and in patients with lung [167,168], osteoarthritis [169], liver, renal [159,170], and myocardial fibrosis [171].

The uptake of [^68^Ga]Ga-FAPI-46 correlates with disease progression in patients with progressive fibrotic ILD, supporting its potential to distinguish active fibrosis from inactive stages, an advantage over conventional CT imaging. Furthermore, FAPI PET imaging shows promise in predicting responses to antifibrotic therapies [167], outperforming traditional [^18^F]FDG-PET which shows limited change post-treatment [172]. [^68^Ga]Ga-FAPI PET also identifies FAP overexpression in renal tissue, serving as a biomarker for early-stage renal fibrosis and post-infarction remodeling [170,173]. Retrospective analyses reveal a significant inverse correlation between renal [^68^Ga]Ga-FAPI-46 uptake and glomerular filtration rate (GFR), linking tracer accumulation to declining kidney function in chronic kidney disease, a relationship not observed with other PET tracers like [^68^Ga]Ga-DOTATOC or [^68^Ga]Ga-PSMA [174]. In cardiac diseases, high intensity cardiac FAPI PET signal correlates with cardiovascular risk factors and metabolic disease, being more increased in patients with multiple risk factors [175]. More recently, in the PROFILE-MI–The FAPI Fibrosis Study (NCT05356923), [^68^Ga]Ga-FAPI-46 has been used to assess myocardial fibroblast activation in 80 subjects after myocardial infarction, demonstrating that it extends beyond the infarct region, decreases slowly with time, persisting for years, and is associated with subsequent left ventricular remodeling [176].

To expand FAP imaging options, an ^18^F-labeled glycosylated FAPI analog, [^18^F]FGIc-FAPI, has been developed, achieving high radiochemical purity (>99%) and molar activity (30–200 GBq/μM) [177]. Although it exhibits somewhat lower FAP binding affinity than [^68^Ga]Ga-FAPI-04, it maintains selective uptake and favorable cellular retention, positioning it as a viable alternative for FAP-targeted PET imaging, with the advantages of ^18^F radionuclide chemistry. PET with the new tracer [^18^F]AlF-ND-bisFAPI has been used for monitoring the progression of liver fibrosis in animal models [178]. Immunohistochemistry showed that the PET signal increased with fibrosis severity, as well as in ex vivo autoradiography of human fibrotic liver sections, where radioactivity increased as fibrosis progressed from mild to severe.

In the context of innovative FAP-targeted CAR-T cell therapy, the new tracer [^18^F]AlF-FAPI-74 has been used to monitor response to treatment in a rodent lung cancer model [179]. [^18^F]AlF-FAPI-74 showed selective retention in FAP^+^ cells in vitro and was able to detect FAP expression on tumor cells as well as FAP^+^ stromal cells in the TME in vivo, providing response biomarkers for FAP-targeted therapy.

In general, a PET imaging approach targeting FAP expressed on activated fibroblasts has a robust potential for non-invasive investigation of many age-related diseases, providing a precise assessment of the severity and stages of fibrosis as well as distribution throughout the organ volume. Ultimately it could be a useful tool for non-invasive monitoring of patients undergoing treatment or effective delivery of innovative therapies directed to FAP.

**Table 1:** Senescence-directed PET radioligands currently used or under evaluation in animal and/or human studies.

PET Radioligand	Molecular Target and Biological Pathways	Target Organs and/or Main Pathologies	Stage of Development	Refs.
[^18^F]FDG	GLUTs (Glucose metabolism)	Multi-organ	Clinically established/Multicenter trials	[47,48,49,50,51,52,53,54,55,56,57]
[^18^F]F-PyGal	β-Gal (DNA damage and oxidative stress)	Osteoarthritis Oncopathologies	Early human feasibility	[64,65]
[^68^Ga]Ga-βGal				
[^18^F]F-FET-SBB	Lipofuscin (Lysosomal degradation of damaged mitochondria)	Oncopathologies	Preclinical only	[71]
[^68^Ga]Ga-DOTA- ECL1i	CCR2 (CCL2/CCR2 axis)	Lung, Liver, Heart, Kidney	Preclinical only	[83,84,85]
[^68^Ga]Ga-DOTA- Pentixafor	CXCR4 (CXCL12/CXCR4 axis)	Brain, Lung, Liver, Heart, Kidney	Clinically established/Multicenter trials	[95,96,97]
[^68^Ga]Ga-DOTA- Siglec-9	VAP-1 (Immune response and evasion)	Rheumatoid Arthritis, Oncopathologies	Early human feasibility	[109,110,111,112,113]
[^18^F]F-FDR-Siglec-9			Preclinical only	
[^68^Ga]Ga-NODAGA- Exendin-4	GLP-1R (Intracellular pathways)	Brain, Heart, Pancreas	Multicenter trials	[143,144,145,146,147,148]
[^68^Ga]Ga-FAPI-04	FAP (Fibroblast activity)	Lung, Liver, Heart, Kidney	Clinically established/ Multicenter trials	[166,167,168,169,170,171,172,173,174,175,176,177,178,179]
[^68^Ga]Ga-FAPI-46				

Abbreviations. Glucose transporters (GLUTs), lysosomal β-galactosidase (β-Gal), C-C chemokine receptor 2 (CCR2), C-X-C motif chemokine receptor 4 (CXCR4), vascular adhesion protein-1 (VAP-1), glucagon-like peptide 1 receptor (GLP-1R), fibroblast activation protein (FAP).

**Table 2:** Current performance of established and emerging senescence-directed PET radioligands.

PET Radioligand	Specificity	Sensitivity/ Affinity	Detection Limit	Biodistribution Variability	Refs.
[^18^F]FDG	Low-moderate (ubiquitous glucose uptake)	High (docking affinity for HXK −14.11 kcal/mol; for GLUT1 −10.37 kcal/mol)	0.3 MBq/kg	High inter-organ uptake (brain, heart, BAT, tumors)	[47,48,49,50,51,52,53,54,55,56,57]
[^18^F]F-PyGal	High	Moderate–high (docking affinity NS)	3.7 MBq/mouse	Mainly osteoarthritic joints, oncopathologies; limited off-target uptake	[64,65]
[^68^Ga]Ga-βGal					
[^18^F]F-FET-SBB	Moderate	High PET sensitivity (docking affinity NS)	NS	High liver accumulation; needs further animal validation	[71]
[^68^Ga]Ga-DOTA- ECL1i	High	High for monocyte/macrophage recruitment (docking affinity −72.3 kJ·mol^−1^)	12 MBq/mouse	Lung, liver, heart, kidney	[83,84,85]
[^68^Ga]Ga-DOTA- Pentixafor	High	High (binding affinity ~24.8 ±2.5 nM)	1.4 to 5.0 MBq/kg	Broad Rapid renal clearance Low non-specific background uptake	[95,96,97]
[^68^Ga]Ga-DOTA- Siglec-9	Moderate–high	High regional uptake at the site of inflamed joints (docking affinity NS)	150 MBq	Rapid blood clearance and renal excretion	[109,110,111,112,113]
[^18^F]F-FDR-Siglec-9					
[^68^Ga]Ga-NODAGA- Exendin-4	High	High (binding affinity 36.0 nM)	105.6 ± 2.3 MBq; peptide dose, 4–7 μg	Rapid blood clearance	[143,144,145,146,147,148]
[^68^Ga]Ga-FAPI-04	High	High (binding affinity 0.04 nM)	1.85–3.7 MBq/kg	Persistent uptake in fibrosis areas	[166,167,168,169,170,171,172,173,174,175,176,177,178,179]
[^68^Ga]Ga-FAPI-46					

Abbreviations. Not specified (NS).

## 3. Discussion

Cellular senescence is a complex biological process integral to aging and the development of numerous age-related diseases. Initially recognized as a tumor-suppressive mechanism that halts uncontrolled cell proliferation, senescence is now understood to have dual roles—both protective and deleterious—in tissue homeostasis. Current evidence supports a paradigm wherein the induction of cellular senescence confers short-term benefits by rendering cells resistant to apoptosis, thus safeguarding them against premature cell death in response to stress. However, this process may exert deleterious effects during later stages, as senescent cells adopt a pro-inflammatory and profibrotic phenotype. The persistence of this state contributes to chronic inflammation and fibrotic tissue remodeling, ultimately compromising tissue homeostasis and facilitating the onset of age-associated pathologies and chronic diseases. The accumulation of senescent cells disrupts normal tissue function through the SASP, characterized by the secretion of inflammatory cytokines and proteases that promote chronic inflammation and contribute to degenerative conditions such as fibrosis, neurodegenerative diseases (e.g., Alzheimer’s and Parkinson’s), cardiovascular disease, and osteoarthritis. Beyond mere growth arrest, senescent cells exhibit metabolic reprogramming, shifting from oxidative phosphorylation to glycolysis, supporting sustained SASP production and highlighting potential metabolic targets for therapeutic intervention. Alterations in lipid metabolism and the accumulation of lipid droplets further emphasize the complex cellular remodeling occurring during senescence.

Detecting and quantifying senescent cells in vivo remains a significant challenge due to their phenotypic and tissue heterogeneity, which complicates the identification of universal biomarkers and the design of effective senotherapies. Moreover, differences between senescence processes in different tissues are unknown. Recent advances have addressed this by developing nuclear feature-based senescence classifiers using machine learning algorithms applied to animal models and human samples, enabling the detection of pathophysiological senescence and facilitating the discovery and validation of potential senotherapeutic agents [180]. The integration of these classifiers with non-invasive imaging modalities, especially PET with novel radiotracers targeting specific aspects of senescence processes offers promising opportunities for quantitative assessment and longitudinal monitoring in living organisms.

Among imaging strategies, PET currently represents the most advanced and clinically translatable modality for senescence imaging due to its exceptional sensitivity, depth penetration, and ability to quantify biomarker expression at picomolar concentrations. PET radioligands such as [^18^F]FPyGal, targeting β-Gal activity as a biomarker of replicative senescence, and [^68^Ga]Ga-FAPI-46, targeting fibroblast activation, have already progressed into clinical research, providing non-invasive, real-time assessment of key senescence hallmarks of premature aging process and fibrosis, respectively. The same vectors used for PET imaging, such as β-Gal or FAPI, may be conjugated to a fluorophore for fluorescence-guided detection. Though valuable in preclinical models or for intraoperative tumor-specific imaging, fluorescent probes present some issues of metabolism and biotoxicity to be addressed, may suffer from high false positive rates and limited tissue penetration, need significant improvement in spatial resolution, and often require specialized equipment, restricting their clinical use [181]. More clinically relevant alternatives to detect senescence—particularly β-Gal activity—include MRI and nuclear magnetic resonance (NMR) approaches [64,182], which, while less sensitive and specific than PET, may be improved through further refinement of contrast agents to enhance senescence-targeting specificity [183]. MRI offers excellent soft-tissue contrast and functional insights such as perfusion or stiffness but lacks the molecular specificity to directly visualize senescence-related markers like β-Gal or SASP factors. Similarly, SPECT imaging can utilize radioligands directed at senescence pathways but typically has lower spatial resolution and quantitative accuracy compared to PET. Nonetheless, its lower cost, greater accessibility, and use of longer half-life isotopes, such as Technetium-99m, make SPECT attractive for exploratory or multicenter studies. Hybrid imaging platforms, such as PET/MRI, combine the molecular precision of PET with the anatomical and functional context of MRI, offering a powerful approach to comprehensively characterize senescence-related processes in vivo [184].

The integration of these imaging technologies—functional (e.g., PET, SPECT), morphological (e.g., MRI, CT), and optical (e.g., lightsheet, super-resolution microscopy), with ‘omics’ phenotyping data and spatial mapping of metabolome, lipidome, and proteome in tissue and cells (e.g., mass spectrometry) supported by computational and AI-driven analysis pipelines [185,186]—will improve our understanding of senescence dynamics and related diseases. These combined approaches are essential to guide the development of targeted senotherapies and translate molecular senescence imaging into routine clinical practice.

PET probes directed to targets with a diagnostic potential across multiple disease settings, i.e., FAP, CXCR4, β-Gal, GLP1-R, rapidly reach clinical translation, especially if they provide cancer targeting, such as FAP in solid tumors, GLP1-R in insulinoma, and CXCR4 in lymphomas. The translational process is slower for radiopharmaceuticals targeting specific pathways not related to oncopathologies, such as Siglec-9 or CCR2. Some technical issues also impact clinical translation of PET probes as proof of target engagement and potential differences between patients and healthy individuals. Finally, a scalable, robust, and reproducible PET radiochemistry must assure high-quality radiopharmaceuticals, complying with a predefined set of specifications for human use to protect patients from unnecessary toxicities and ensure the best possible diagnostic accuracy.

Additional challenges may derive from emerging innovative treatment options for age-related disorders such as nanosystems, vaccines, CAR-T cell therapies directed to specific targets, requiring accurate, non-invasive and quantitative imaging methods to assess effective treatment delivery and monitor efficacy.

The convergence of machine learning, advanced molecular imaging, and standardized experimental protocols paves the way for more precise and personalized senotherapeutic strategies, as demonstrated in multiple recent clinical trials for the treatment of osteoarthritis (OA) (NCT04210986 enrolling 75 patients with mild-to-moderate OA; NCT03513016 including 78 subjects with painful knee OA), pulmonary fibrosis (NCT02874989: 26 patients with idiopathic pulmonary fibrosis; NCT00764309: 47 patients with scleroderma pulmonary interstitial fibrosis), mild and severe AD and PD (NCT02256306: 18 patients with mild-to-moderate AD; NCT03765762: 26 patients with severe AD; NCT04063124: 5 patients with AD progression; NCT04369430: 110 subjects with Parkinson’s Disease on stable dopaminergic treatment), frailty and bone health (NCT02652052: 9 hematopoietic stem cell transplant survivors; NCT03430037: 40 elderly women with gait disturbance; NCT02874924: 34 elderly patients; NCT04313634: 60 postmenopausal women), and liver fibrosis (NCT06160271: 72 patients with biopsy for suspected or proven nonalcoholic steatohepatitis), and they could also have a place in combination with oncological treatment to improve efficacy [187,188].

These innovations hold the potential not only to deepen our understanding of senescence biomechanisms but also to translate into clinical applications that effectively mitigate the burden of age-related diseases.

## 4. Conclusions

Cellular senescence plays a pivotal role in driving many chronic diseases, with its complex interplay of oxidative stress, inflammation, and altered proteostasis contributing to tissue dysfunction and aging. The accumulation of senescent cells, characterized by their distinct morphologies and functional profiles, poses significant challenges in understanding and managing these conditions. Currently, there are no available global senescence markers which could offer promising avenues for early diagnosis and therapeutic intervention.

The identification of precise targets and next-generation imaging techniques such as PET with novel radioligands is revolutionizing our ability to detect and monitor senescence phenotypes in vivo. While PET imaging with established radioligands such as FAPI, GLP-1R, and CXCR4 is already advancing clinical applications, the development of novel tracers specifically targeting senescence represents a longer-term opportunity. By integrating molecular insights with advanced imaging and biomarker development, researchers are poised to refine therapeutic approaches, ultimately mitigating the senescence process and improving clinical benefit.

## Data Availability

No new data have been generated in this article.
